# *KRT13* is upregulated in pancreatic cancer stem-like cells and associated with radioresistance

**DOI:** 10.1093/jrr/rrac091

**Published:** 2023-01-04

**Authors:** Wataru Takenaka, Yuhki Yokoyama, Katsuya Ikehata, Shihori Kouda, Haruka Hirose, Kazumasa Minami, Yoshinosuke Hamada, Seiji Mori, Masahiko Koizumi, Hirofumi Yamamoto

**Affiliations:** Department of Medical Physics and Engineering, Division of Health Sciences, Graduate School of Medicine, Osaka University, 1-7 Yamadaoka, Suita city, Osaka, 565-0871, Japan; Department of Molecular Pathology, Division of Health Sciences, Graduate School of Medicine, Osaka University, 1-7 Yamadaoka, Suita city, Osaka, 565-0871, Japan; Department of Medical Physics and Engineering, Division of Health Sciences, Graduate School of Medicine, Osaka University, 1-7 Yamadaoka, Suita city, Osaka, 565-0871, Japan; Department of Molecular Pathology, Division of Health Sciences, Graduate School of Medicine, Osaka University, 1-7 Yamadaoka, Suita city, Osaka, 565-0871, Japan; Department of Systems Biology, Graduate School of Medicine, Nagoya University, 65 Tsurumai-cho, Showa-ku, Nagoya city, Nagoya, 466-8550, Japan; Department of Medical Physics and Engineering, Division of Health Sciences, Graduate School of Medicine, Osaka University, 1-7 Yamadaoka, Suita city, Osaka, 565-0871, Japan; Department of Health Economics and Management, Graduate School of Medicine, Osaka University, 1-7 Yamadaoka, Suita city, Osaka, 565-0871, Japan; Department of Pediatric Dentistry, School of Dentistry, Osaka Dental University, 8-1 Kuzuhahanazono-cho, Hirakata city, Osaka, 573-1121, Japan; Department of Medical Technology, Faculty of Health Sciences, Morinomiya University of Medical Sciences, 1-26-16 Nankokita, Suminoe-ku, Osaka city, Osaka, 559-8611, Japan; Department of Medical Physics and Engineering, Division of Health Sciences, Graduate School of Medicine, Osaka University, 1-7 Yamadaoka, Suita city, Osaka, 565-0871, Japan; Department of Molecular Pathology, Division of Health Sciences, Graduate School of Medicine, Osaka University, 1-7 Yamadaoka, Suita city, Osaka, 565-0871, Japan; Department of Surgery, Gastroenterological Surgery, Graduate School of Medicine, Osaka University, 2-2 Yamadaoka, Suita city, Osaka, 565-0871, Japan

**Keywords:** *KRT13*, cancer stem-like cell (CSC), radioresistance, pancreatic cancer

## Abstract

Pancreatic cancer is one of the most aggressive cancers and the seventh leading cause of cancer-associated death in the world. Radiation is performed as an adjuvant therapy as well as anti-cancer drugs. Because cancer stem-like cells (CSCs) are considered to be radioresistant and cause recurrence and metastasis, understanding their properties is required for the development of novel therapeutic strategies. To investigate the CSC properties of pancreatic cancer cells, we used a pancreatic CSC model, degron (++) cells, which have low proteasome activity. Degron (++) cells displayed radioresistance in comparison with control cells. Using Ribonucleic acid (RNA) sequencing, we successfully identified *KRT13* as a candidate gene responsible for radioresistance. Knockdown of *KRT13* sensitized the degron (++) cells to radiation. Furthermore, a database search revealed that *KRT13* is upregulated in pancreatic cancer cell lines and that high expression of *KRT13* is associated with poorer prognosis. These results indicate that a combination therapy of *KRT13* knockdown and radiation could hold therapeutic promise in pancreatic cancer.

## INTRODUCTION

Pancreatic cancer is one of the most aggressive cancers and its prognosis is extremely poor worldwide [1]. Radiotherapy is an option for adjuvant therapy for pancreatic cancer [2, 3]. Although several irradiation methods with high accuracy, such as intensity-modulated radiation therapy, equipment and treatment planning systems have been developed, some patients still experience recurrence.

One possible reason for this problem is the existence of cancer stem-like cells (CSCs). Many studies have shown that CSCs constitute a small population existing in the tumor and have the capacity for self-renewal, high tumorigenicity and resistance to conventional cancer therapies such as radiotherapy and chemotherapy [4–8]. The CSC population is therefore considered to be the source of recurrence and metastasis. Although a number of studies have been conducted to elucidate the properties of CSCs and establish a new treatment strategy for targeting them, as yet no therapeutic targeting CSC is available [9].

One difficulty in analyzing the properties of CSCs is the isolation of the CSC population from tumors. To acquire the CSC population, cell surface markers are often used. In pancreatic cancer, several cell surface markers, such as CD133, CD24, CD44 and epithelial specific antigen, are reported to be CSC markers [10]. In addition to the cell surface markers, low proteasome activity is reportedly a hallmark of CSCs. The proteasome is an enzymatic protein complex that degrades unnecessary or damaged proteins. Cancer cells have high proteasome activity; therefore, proteasome inhibitors such as bortezomib and carfilzomib are used clinically as anti-cancer drugs [11]. On the other hand, it has been demonstrated that cancer cells with low proteasome activity have CSC properties, including radioresistance [12, 13]. CSCs can thus be purified on the basis of this difference in proteasome activity.

To assess their proteasome activity, cells are engineered to stably express green fluorescent protein (ZsGreen) fused to the degron sequence of ornithine decarboxylase (ODC), which is directly recognized by the proteasome. Because ZsGreen accumulates in cells such as CSCs that have low proteasome activity, this degron system enables real-time monitoring and cell sorting of CSCs without any treatment. In addition, this system is useful for multiple cancer types because proteasome activity is a common biologic property [14–21]. Collectively, this method is better than cell surface marker methods, which depend on cancer type and require staining.

In this study, we generated PANC-1 cells stably expressing ZsGreen fused to the ODC degron to isolate the CSC population and identify the genes responsible for radioresistance. We found that *KRT13* is upregulated in pancreatic CSCs and associated with radioresistance. Database analysis revealed that *KRT13* is highly expressed in pancreatic cancer cell lines and that high expression of *KRT13* is associated with poorer prognosis. Taken together, these results suggest that *KRT13* knockdown in combination with radiotherapy could be a new strategy for treating pancreatic cancer.

## MATERIALS AND METHODS

### Generation of a pancreatic CSC model

Pancreatic CSC model was generated as described previously [22, 23]. Briefly, the retroviral expression vector pQCXIN-ZsGreen-cODC, which contains ZsGreen gene fused to the carboxyl-terminal degron sequence of ODC was transfected into Platinum retroviral packaging cells and the retrovirus collected from the supernatant was infected to human pancreatic cancer cell line PANC-1. pQCXIN-ZsGreen-cODC was kindly provided by Dr Frank Pajonk (Jonsson Comprehensive Cancer Center, UCLA, CA, USA). We defined this infected PANC-1 cell line as degron (−). Because degron (−) contains few ZsGreen-positive cells (<1%), ZsGreen-positive degron (−) cells could be repeatedly sorted out by using an SH800Z cell sorter (SONY, Tokyo, Japan) and cultured. This allowed us to obtain a concentrated cell population containing more than 40% ZsGreen-positive cells (degron (+)). For the *in vitro* experiment, the 50% of cells that were the most ZsGreen positive in this population were used as a CSC model, defined as degron (++). All assays (irradiation, small interfering RNA (siRNA) transfection) were performed 48 hours after cell sorting in order to reduce cell damage. Cells were cultured in Dulbecco’s Modified Eagle’s Medium (Sigma-Aldrich, St. Louis, MO, USA) with low glucose, 10% fetal bovine serum (Sigma-Aldrich) and G418 (Roche, Basel, Switzerland) for selection at 37°C in a humidified atmosphere of 5% CO_2_.

### Irradiation

Cells in a culture flask were irradiated with a cesium-137 gamma-ray irradiator, (Gammacell 40 Exactor) at our facility, at a dose rate of approximately 0.81 Gy/min.

### Colony formation assay

Immediately after irradiation, cells were trypsinized and seeded at 1000 or 5000 cells into 6-cm dishes. Fourteen days later, these cells were fixed with 4% paraformaldehyde and stained with a crystal violet solution. After staining, the colonies were counted and cell viability was calculated.

### Flow cytometry analysis

Apoptotic cells were detected by using a GFP-Certified Apoptosis/Necrosis Detection Kit (Enzo Life Sciences, East Farmingdale, NY, USA) according to the manufacturer’s instructions. Apoptotic cells were stained with annexin V–EnzoGold and/or 7-amino-actinomycin D (7AAD). For cell labeling to evaluate the cell surface expression of CD44 variant 9 (CD44v9) and CD133, primary antibody to CD44v9 (clone RV3, rat-IgG monoclonal, Cosmo Bio, Tokyo, Japan) and CD133 (clone AC133, mouse-IgG monoclonal, Miltenyi Biotec, Cologne, German) were used. As a secondary antibody, PE conjugated anti rat-IgG (clone RG7/1.30, BD Bioscience, NJ, USA) and APC conjugated anti mouse-IgG (clone X57, Miltenyi Biotec) were used. The analysis was performed by using an SH800Z Cell Sorter.

### Sphere formation assay

Cells were seeded in 96-Well Clear Ultra Low Attachment Microplates (Corning, Inc., Corning, NY, USA) at a density of 500 cells per well. For siRNA experiment, cells were seeded after 48 hours of siRNA treatment. The cells were cultured in DMEM serum-free medium (Thermo Fisher Scientific, Waltham, MA, USA) supplemented with 20 ng/ml epithelial growth factor, 10 ng/ml basic fibroblast growth factor-2 (PeproTech, Inc., Cranbury, NJ, USA), 100 U/ml penicillin and 100 μg/ml streptomycin, following our previous article [23]. Cells were cultured in a humidified incubator at 37°C and 5% CO_2_. Images were captured by a bright field light microscope (CKX53; Olympus Corporation, Tokyo, Japan) with Visualix camera (Visualix, Kobe, Japan). The number of spheroids was counted larger than 100 μm in size manually on days 5 after seeding.

### Reverse transcription quantitative PCR analysis

Total RNA was purified from cultured cells using TRIzol Reagent (Thermo Fisher Scientific), and complementary DNA was generated from 2.0 μg total RNA using High Capacity cDNA Reverse Transcription kit (Thermo Fisher Scientific) following to the manufacturer’s instructions. Quantitative polymerase chain reaction (qPCR) was conducted by using THUNDERBIRD Next SYBR qPCR Mix (TOYOBO, Osaka, Japan). Relative expression was quantified using the 2^−ΔΔ^Ct method. The expression level of the target gene was normalized by GAPDH mRNA expression.

Primers are shown below:

GAPDH F [5′- CAACTACATGGTTTACATGTTC], R [5′- GCCAGTGGACTCCACGAC].

KRT13 F [5′- CCCCAGGCATTGACCTGAC], R [5′- GTGTTGGTAGACACCTCCTTG].

### RNA sequence data

To identify candidate radioresistance genes, RNA sequence analysis was performed as previously described [24]. Gene expression data were analyzed using fold change and fragments per kilobase of exon per million reads mapped (FPKM). Fold change means the ratio of gene expression between degron (−) and degron (++) cells. FPKM means the absolute value of gene expression used in RNA sequence analysis. The raw data were deposited in the NCBI Gene Expression Omnibus database under accession number GSE212883. For gene enrichment analysis, we used KEGG pathway database (https://www.genome.jp/kegg/pathway.html). In addition, we performed GSEA with PreRank option to input the whole list of genes. We used the gene sets from the Broad Institute’s Molecular Signature Database. False discovery rate (FDR) q-value <0.05 was considered as the threshold for significance.

### siRNA transfection

Cells were transfected with siRNA against *KRT13* (si*KRT13*) (FlexiTube siRNA, Qiagen, Hilden, Germany) and negative control siRNA (siNC) (GeneDesign, Osaka, Japan) by the use of Lipofectamine RNAiMAX (Thermo Fisher Scientific) according to the manufacturer’s instructions. Cells were irradiated 24 hours after transfection.

### Data analysis

The Cancer Cell Line Encyclopedia (CCLE) database (https://portals.broadinstitute.org/ccle) was used for the analysis of mRNA expression in the multiple cancer cell lines. The survival analysis was performed with the cancer genome atlas (TCGA) database and the Oncolnc tool (http://www.oncolnc.org/) [25].

### Statistics

Data are shown as mean ± SD. The data were compared by using the Student t-test. A value of *P* < 0.05 was considered statistically significant. All statistical analyses were performed using Microsoft Excel.

## RESULTS

### Generation of the pancreatic CSC model

**Fig. 1:**
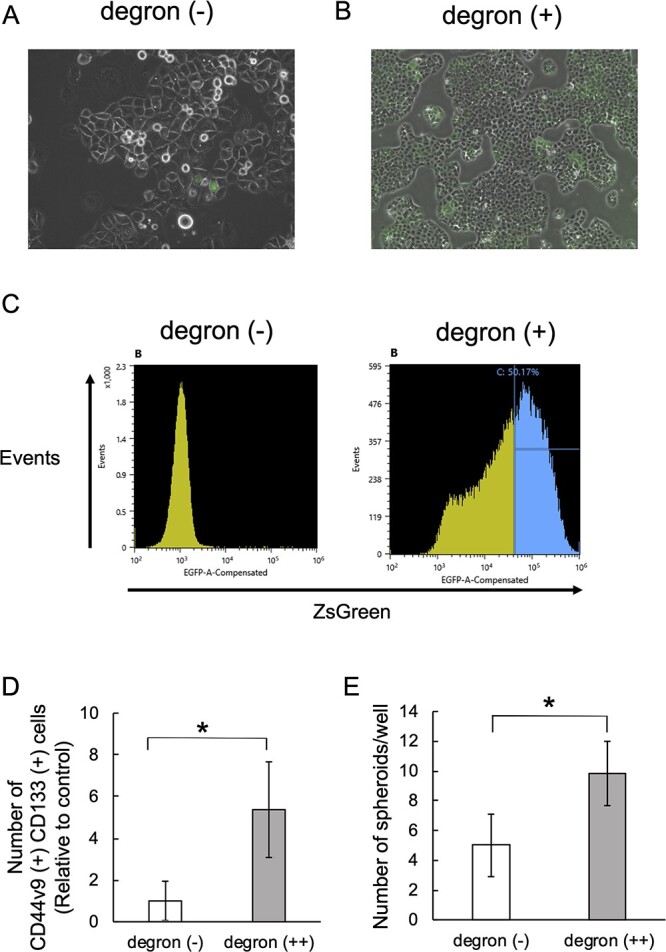
Generation of a pancreatic CSC model by use of the ODC-degron system. A. Photomicrograph of degron (−) cells. The percentage of ZsGreen-positive cells was less than 1%. B. Photomicrograph of degron (+) cells. The percentage of ZsGreen-positive cells was more than 40%. C. The expression levels of ZsGreen in the degron (−) and degron (+) cell populations. The 50% of cells with the highest ZsGreen expression (blue color) were defined as degron (++) cells and used for the subsequent experiments. D. The number of CD44v9 and CD133 double positive population in degron (−) and degron (++) cells. The number relative to the control cells (degron (−)) are shown. Each bar represents the mean ± standard deviation (^*^  *P* < 0.05). E. The number of spheroids in degron (−) and degron (++). Spheroids (>100 um) were counted on day 5 after seeding. Each bar represents the mean ± standard deviation (^*^  *P* < 0.05).

We generated PANC-1 cells stably expressing ZsGreen fused to the ODC degron to visualize CSCs as cells with low proteasome activity. In this cell line, fewer than 1% of the cells were ZsGreen positive; therefore, we defined this cell line as degron (−) cells (Fig. 1A and C). This percentage was reasonable because the CSC population in the tumor is rare, but it might have been difficult to accurately analyze the properties of CSCs with this model. Therefore, we concentrated the ZsGreen-positive cell population by repeated FACS and culturing. We eventually obtained the degron (+) cell line, which contains more than 40% ZsGreen-positive cells (Fig. 1B and C right). To further concentrate the CSC population, the 50% of cells that were the most ZsGreen positive (defined as degron (++)) were used for the subsequent experiments (Fig. 1C blue color). We confirmed that degron (++) cells showed high expression of the cell surface CSC markers (CD44v9 and CD133) and increased the number of spheroids compared with degron (−) cells (Fig. 1D and E).

### Degron (++) cells displayed higher radioresistance

**Fig. 2:**
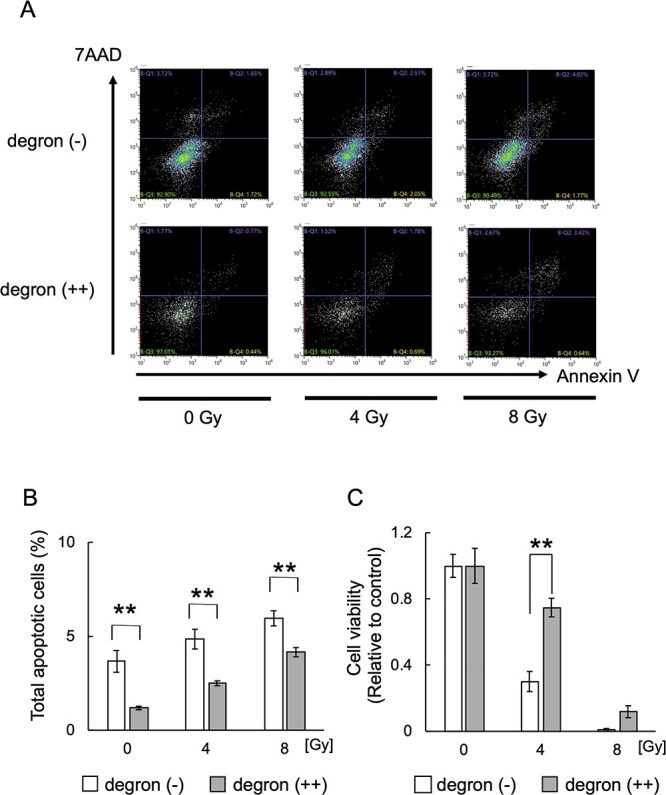
Radioresistance was higher in degron (++) cells than in degron (−) cells. A. Flow cytometry analysis of annexin V–positive (X axis) and 7-AAD–positive (Y axis) cells 72 hours after irradiation of degron (−) and degron (++) cells. B. Bar chart of the apoptotic cells (annexin V–positive cells) shown in Fig. 2A. Each bar represents the mean ± standard deviation (^**^  *P* < 0.01). C. Cell viability of degron (−) and degron (++) cells fourteen days after irradiation. The values relative to the control cells (no irradiation) is shown. Each bar represents the mean ± standard deviation (^**^  *P* < 0.01).

To investigate the radioresistance of degron (++) cells, we performed an annexin V assay and found that the number of apoptotic cells was lower in degron (++) cells than in degron (−) cells 72 hours after irradiation at 4 and 8 Gy (Fig. 2A and B). We also performed a colony formation assay to assess long-term cell survival after irradiation. After irradiation, the viability of degron (++) cells was significantly higher than that of degron (−) cells (Fig. 2C). These results indicate that the degron (++) cells were more radioresistant than the degron (−) cells.

### *KRT13* gene expression was upregulated in degron (++) cells

To identify candidate genes associated with radioresistance in degron (++) cells, RNA sequence analysis was performed to compare gene expression in degron (++) cells with that in degron (−) cells. Among over 20 000 genes analyzed, the top 10 most upregulated genes in degron (++) cells are shown in Table 1. We focused on *KRT13* as a candidate gene responsible for radioresistance in pancreatic CSC because an association of some KRT family genes, such as *KRT19*, with the stemness of normal and cancer cells has been reported [26–29].

**Table 1:** Gene list that is upregulated in degron (++) cells

**Gene**	**Fold change**	**FPKM**		**Description**
		**Degron (++)**	**Degron (−)**	
MMP12	36.02	39.69	1.17	matrix metallopeptidase 12 (macrophage elastase)
COL4A4	32.64	11.03	0.34	collagen, type IV, alpha 4
TMPRSS3	29.61	8.26	0.33	transmembrane protease, serine 3
TMEM125	28.35	8.65	0.37	transmembrane protein 125
STEAP4	23.52	9.08	0.39	STEAP family member 4
*KRT13*	22.24	83.22	3.89	keratin 13
TJP3	22.09	8.12	0.38	tight junction protein 3
TNFRSF11B	20.53	91.00	4.48	tumor necrosis factor receptor superfamily, member 11b
PSG5	20.21	7.46	0.40	pregnancy specific beta-1-glycoprotein 5
MUC16	19.94	6.88	0.34	mucin 16, cell surface associated

### *KRT13* knockdown decreased radioresistance and stem-like cell property in degron (++) cells

**Fig. 3:**
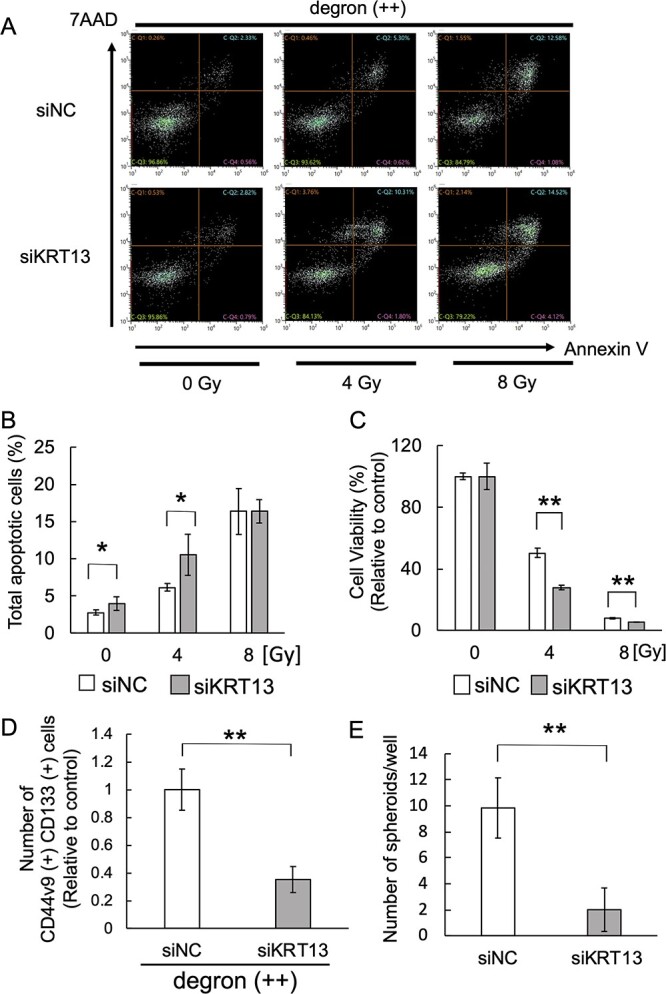
*KRT13* knockdown decreased radioresistance and stem-like cell property in degron (++) cells. A. Flow cytometry analysis of annexin V–positive (X axis) and 7-AAD–positive (Y axis) cells 72 hours after the irradiation of siNC- and si*KRT13*-treated degron (++) cells. B. Bar chart of the apoptotic cells (annexin V–positive cells) shown in Fig 3A. Each bar represents the mean ± standard deviation (^*^  *P* < 0.05). C. Viability of siNC- and si*KRT13*-treated degron (++) cells fourteen days after irradiation. Values relative to the control cells (no radiation) are shown. Each bar represents the mean ± standard deviation (^**^  *P* < 0.01). D. The number of CD44v9 and CD133 double positive population in siNC- and si*KRT13*-treated degron (++) cells. The number relative to the control cells (siNC-treated) are shown. Each bar represents the mean ± standard deviation (^**^  *P* < 0.01). E. The number of spheroids in siNC- and si*KRT13*-treated degron (++) cells. Spheroids (>100 um) were counted on day 5 after seeding. Each bar represents the mean ± standard deviation (^**^  *P* < 0.01).

To investigate whether *KRT13* is related to the radioresistance in degron (++) cells, we performed the knockdown experiment. We verified that si*KRT13* treatment decreased the mRNA expression of *KRT13* in degron (++) cells (Supplementary Fig. 1). Knockdown of *KRT13* induced apoptosis in degron (++) cells even without irradiation, and this effect was further enhanced by irradiation at 4 Gy, but not enhanced at 8 Gy (Fig. 3A and B). The colony formation assay showed that the viability of degron (++) cells after irradiation was significantly decreased in si*KRT13*-treated cells relative to that of cells treated with negative control siRNA (Fig. 3C). Furthermore, we showed that si*KRT13* treatment decreased the cell surface CSC markers (CD44v9 and CD133) and the number of spheroids in degron (++) cells (Fig. 3D and E). These results suggest that *KRT13* is related to the radioresistance and stemness of pancreatic cancer cells.

### *KRT13* is upregulated in pancreatic cancer and associated with poor prognosis

**Fig. 4:**
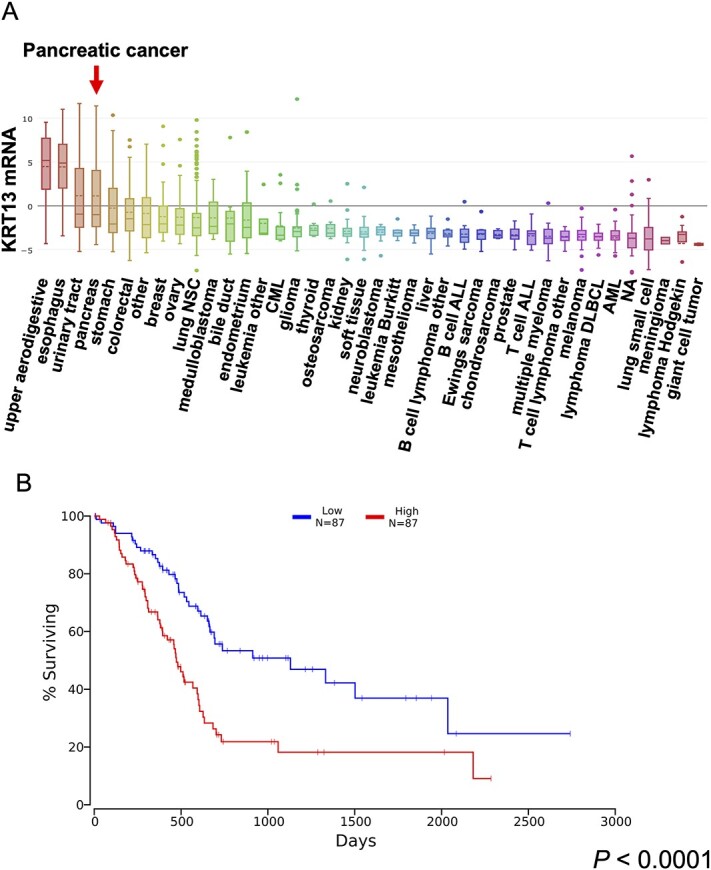
*KRT13* is upregulated in pancreatic cancer cell lines, and high *KRT13* expression is associated with poorer prognosis in pancreatic cancer. A. *KRT13* expression levels in multiple cancer cell lines registered in the CCLE database. B. Patient survival in *KRT13*^low^ and *KRT13*^high^ expression groups in pancreatic cancer. The analysis was performed by using the OncoLnc web tool. *P* < 0.0001.

To investigate the clinical significance of *KRT13* in pancreatic cancer, we analyzed a publicly available database and found that *KRT13* expression is upregulated in pancreatic cancer cell lines (Fig. 4A; rank no. 4 among the multiple cancer types). Furthermore, TCGA survival data showed that high *KRT13* expression is associated with poor prognosis in patients with pancreatic cancer (Fig. 4B; *P* < 0.0001).

## DISCUSSION

In this study, we aimed to discover the gene responsible for radioresistance in pancreatic CSCs. To achieve this purpose, we used a ZsGreen-ODC degron fusion reporter system [12]. This system enables the visualization of cells with low proteasome activity, which has been considered a property of CSCs, so that the CSC population can be sorted and concentrated by FACS. Adikrisna *et al.* revealed that ZsGreen highly positive PANC-1 cell population showed the increased sphere formation, chemoresistance, asymmetric cell division and *in vivo* tumorigenesis, which represent the stem-like characteristics, compared with the control cells [22]. In addition, we previously showed that ZsGreen highly positive PANC-1 cell population were resistant to several microRNA treatments compared with control cells [23]. Using this approach, we acquired the degron (+) cell line and confirmed that the further concentrated degron (++) cells displayed greater radioresistance and increased expression of the cell surface CSC markers (CD44v9 and CD133) and the number of spheroids compared with the non-concentrated degron (−) cells (<1% ZsGreen-positive cells). This indicates that we successfully generated a CSC model for pancreatic cancer.

We performed RNA sequence analysis to identify the gene responsible for radioresistance in pancreatic CSCs. In addition to identify the gene responsible for radioresistance, we performed gene enrichment analysis by using public database, Kyoto Encyclopedia of Genes and Genomes (KEGG) and Gene Set Enrichment Analysis (GSEA). Gene enrichment analysis using KEGG pathway database showed that 421 upregulated genes (Fold change >5 and *P* < 0.01) in degron (++) cells compared with degron (−) cells were enriched in Axon guidance, ECM-receptor interaction and PI3K/AKT signaling pathway (Supplementary Table 1). On the other hand, we found the significant enrichment with 44 gene sets including KRAS dependency signature in degron (++) cells by GSEA (Supplementary Table 2, Supplementary Fig. 2). Further investigation is needed to clarify whether these pathways are related to the radioresistance and stemness in pancreatic cancer cells.

As a result of RNA sequence analysis, several genes were identified whose expression in degron (++) cells was much higher than in degron (−) cells. These included matrix metalloproteinase-12 (*MMP12*), Collagen Type IV Alpha 4 Chain (*COL4A4*) and Transmembrane protease serine 3 (*TMPRSS3*). It is reported that MMP12 is upregulated by ultra violet (UV) irradiation in human skin [30]. COL4A4 is one of the components of type IV collagen. There are no reports of a direct relationship between radiation and this gene. However, it is known that radiation induced TGF-β activation [31]. And there is a positive correlation between TGF-β expression and enhanced collagen production. TMPRSS3 is a membrane-bound serine protease overexpressed in pancreatic cancer. This proteinase is of importance for processes involved in tumor invasion [32]. It also reported in another article that it is related with radioresistance [33]. In the present study, we focused on *KRT13* as a candidate radioresistance gene because certain keratins (KRTs) are reportedly shown to have cancer stem-like properties, including *KRT19* in hepatocellular carcinoma and colorectal cancer, *KRT6* in lung cancer and *KRT17* in cervical cancer [26–28, 34]. Accordingly, we took notice of *KRT13* among several candidates listed in Table 1. During preparation of this manuscript, other investigators reported that *KRT13* promotes the stemness of breast cancer cells and *KRT13* is associated with radioresistance and stemness in squamous cell carcinoma cells, which further emphasized the importance of *KRT13* in cancer stem cells [35, 36].

The KRTs are a family of intermediate filament proteins that constitute the cytoskeleton of epithelial cells. KRTs are often used in histopathology to determine cancer cell origins and aid the prognosis of several malignancies [37, 38]. Although the maintenance of cellular structure is well-known function of KRTs, recent studies have suggested that KRTs are involved in functions such as apoptosis, cell growth and cell motility in cancer. For example, abnormal expression of *KRT8* and *KRT18* is related to tumor progression and invasion in squamous carcinomas [39]. *KRT17* has been identified as an oncogene in cervical cancer [40]. Regarding CSCs, high *KRT19* expression is correlated with cancer stemness and radioresistance in hepatocellular carcinoma and colorectal cancer [27, 28]. In contrast, cells expressing lower levels of *KRT19* displayed increased level of stem cell markers, colony-formation activity and drug resistance in breast cancer [41, 42]. In our study, *KRT19* expression was not increased in degron (++) cells (data not shown).

*KRT13* is a type I keratin expressed in the suprabasal layers of noncornified squamous epithelia such as those of the oral cavity, tonsils and esophagus [43]. In cancer, diverse clinical relevance and functions of *KRT13* have been reported. *KRT13* expression is decreased in oral and cervical cancer tissue [44, 45], and the gene is epigenetically silenced in an oral squamous cell carcinoma cell line and in invasive bladder cancer tissue [46, 47]. On the other hand, *KRT13*-overexpressing prostate cancer cell lines are reported to be highly migratory, with high expression of genes related to the epithelial-mesenchymal transition and CSCs [48]. Another study showed that *KRT13* expression is increased in the tumor zone with a higher degree of stemness in prostate cancer [49]. Notably, a recent study showed that enhanced *KRT13* expression is associated with radioresistance in squamous carcinoma cells, consistent with our current finding in pancreatic cancer [36]. We showed that si*KRT13* treatment significantly enhanced the radiosensitivity of degron (++) cells after irradiation at 4 Gy, but not enhanced at 8 Gy. One possible reason for this result is that 8 Gy irradiation is lethal to cancer cells, even CSCs. To our knowledge, this is the first study to demonstrate the function and clinical relevance of *KRT13* in pancreatic cancer.

Our results indicate that a combination therapy of *KRT13* knockdown and radiation could be an effective strategy in pancreatic cancer. Nucleic acid–based medicines such as antisense oligonucleotides, siRNAs and miRNAs are anticipated as next-generation therapeutics. However, there are several clinical challenges. For example, nucleic acids are unstable in the blood stream and accumulate in the liver and kidney. Effective tumor targeting thus requires a drug delivery system. We developed the super carbonate apatite (sCA) nanoparticle, a pH-sensitive drug delivery system, and showed that siRNA and miRNA can be stably and efficiently transferred to tumors by sCA without any toxicity [50–52]. Although further investigation is required, the combination therapy of sCA-siRNA for *KRT13* and radiation may be a promising therapeutic approach in pancreatic cancer.

## Supplementary Material

Supplementary_Fig_1_rrac091

Supplementary_Fig_2_rrac091

Supplementary_Table1_rrac091

Supplementary_Table2_rrac091

Supplementary_Figures_in_MS_word_file_rrac091
