# Supplementary figures and images for: KRT13 is upregulated in pancreatic cancer stem-like cells and associated with radioresistance
Source: J Radiat Res. 2023 Jan 4;64(2):284–93. doi: 10.1093/jrr/rrac091 (PMC10036105; doi:10.1093/jrr/rrac091)

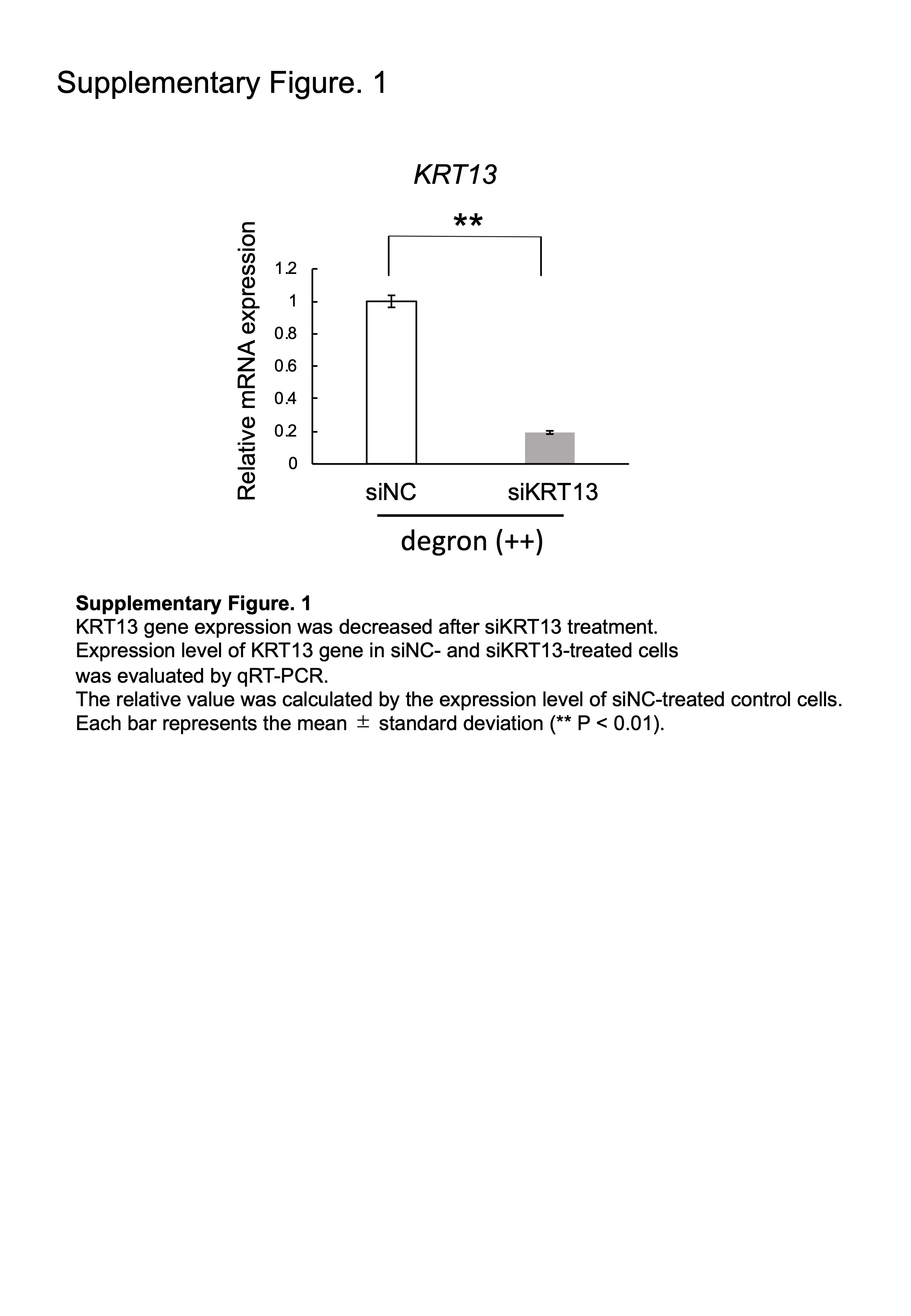

Supplement: Supplementary_Fig_1_rrac091 [file supplementary_fig_1_rrac091.jpeg]

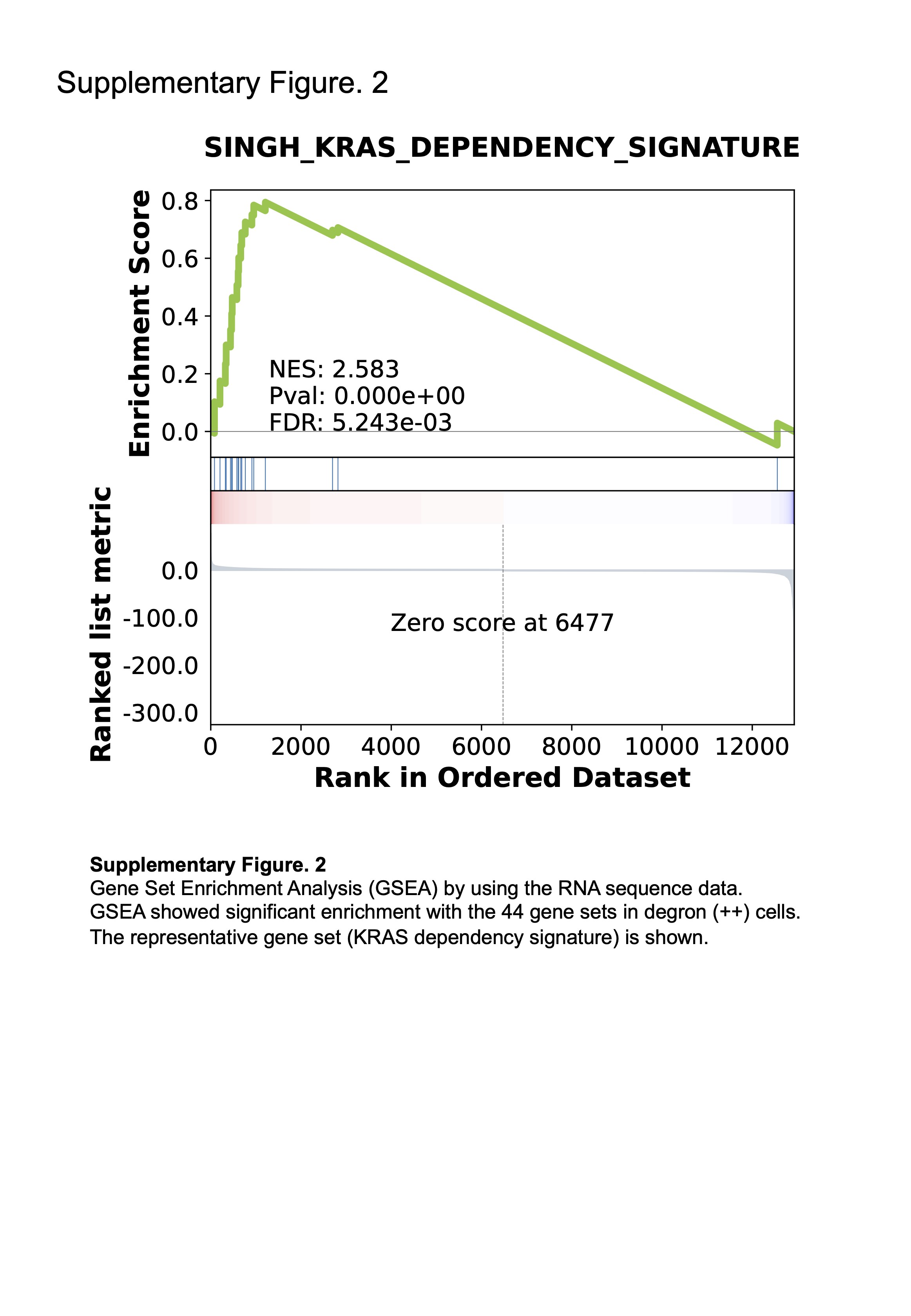

Supplement: Supplementary_Fig_2_rrac091 [file supplementary_fig_2_rrac091.jpeg]

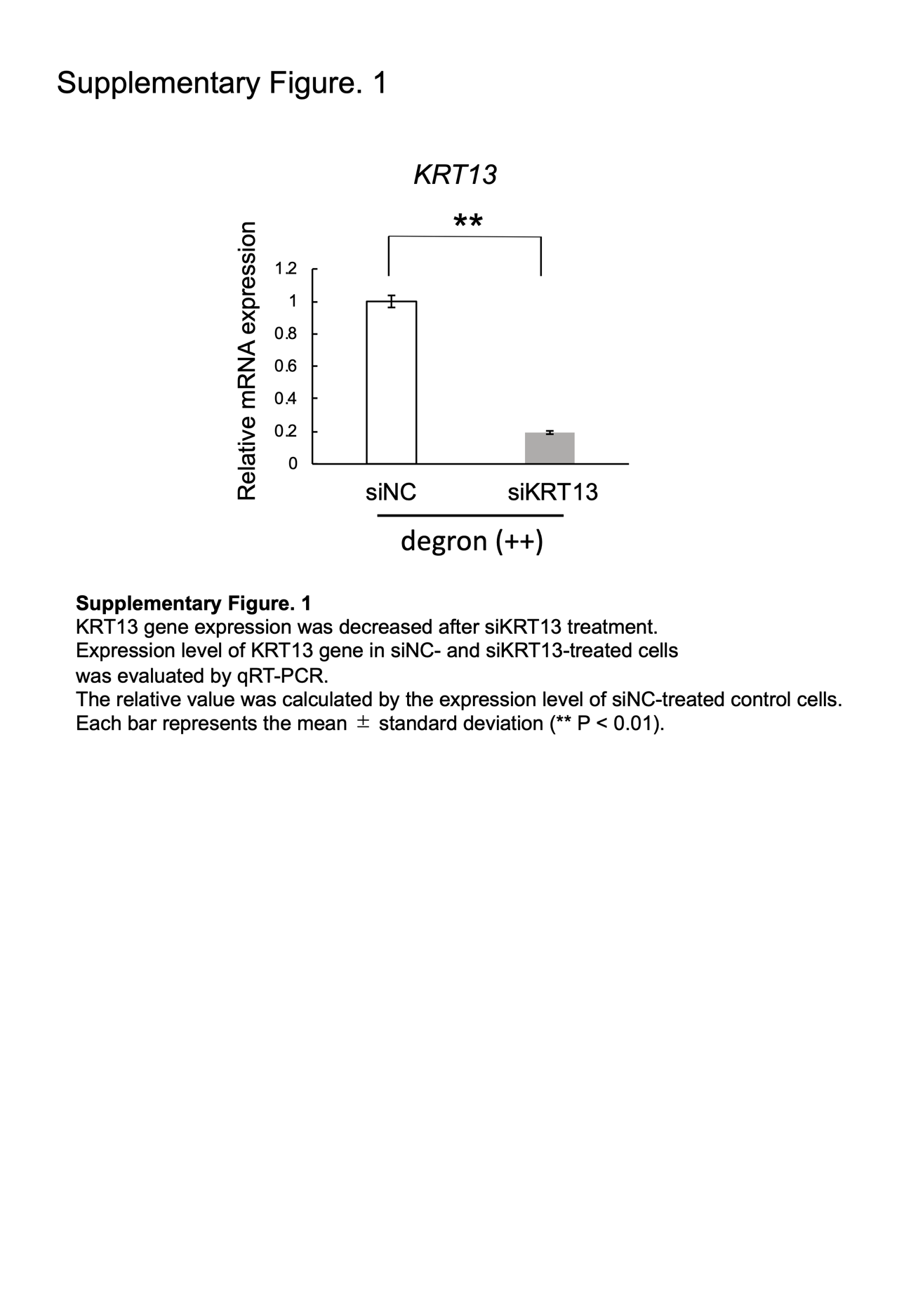


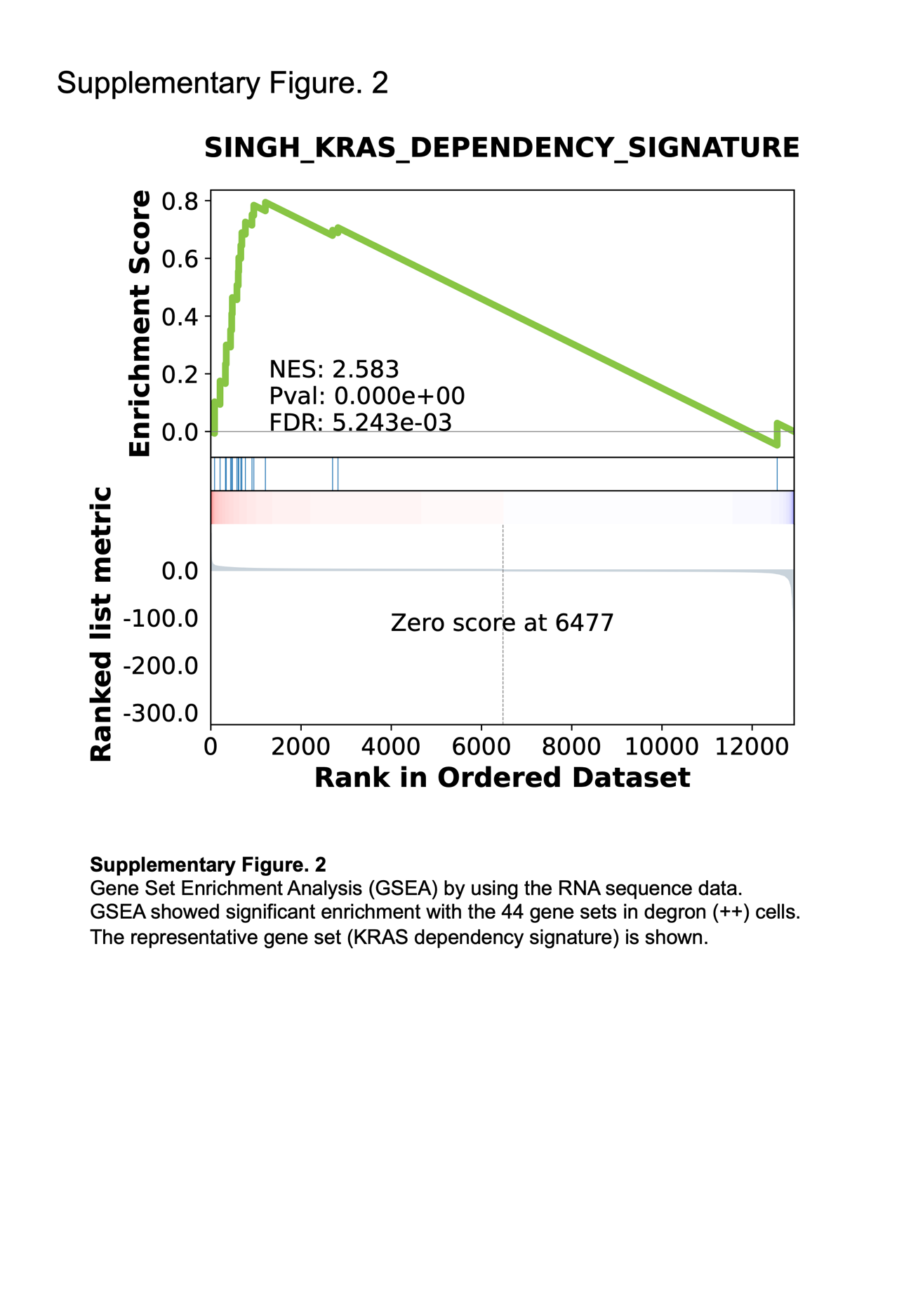

Supplement: Supplementary_Figures_in_MS_word_file_rrac091 [file supplementary_figures_in_ms_word_file_rrac091.docx]
